# PROLONG: a cluster controlled trial to examine identification of patients with COPD with poor prognosis and implementation of proactive palliative care

**DOI:** 10.1186/1471-2466-14-54

**Published:** 2014-04-02

**Authors:** Ria G Duenk, Yvonne Heijdra, Stans C Verhagen, Richard PNR Dekhuijzen, Kris CP Vissers, Yvonne Engels

**Affiliations:** 1 Department of Anesthesiology, Pain and Palliative Medicine, Radboud University Nijmegen Medical Centre, PO Box 9101, Nijmegen 6500 HB, the Netherlands; 2 Department of Lung Diseases, Radboud University Nijmegen Medical Centre, PO Box 9101, Nijmegen 6500 HB, the Netherlands

**Keywords:** COPD, Exacerbation, Indicators, Prognosis, Proactive palliative care, Quality of life

## Abstract

**Background:**

Proactive palliative care is not yet common practice for patients with COPD. Important barriers are the identification of patients with a poor prognosis and the organization of proactive palliative care dedicated to the COPD patient. Recently a set of indicators has been developed to identify those patients with COPD hospitalized for an acute exacerbation who are at risk for post-discharge mortality. Only after identification of these patients with poor prognosis a multi disciplinary approach to proactive palliative care with support of a specialized palliative care team can be initiated.

**Methods/Design:**

The PROLONG study is a prospective cluster controlled trial in which 6 hospitals will participate. Three hospitals are selected for the intervention condition based on the presence of a specialized palliative care team. The study population consists of patients with COPD and their main informal caregivers. Patients will be included during hospitalization for an acute exacerbation. All patients in the study receive standard care (usual care). Besides, patients in the intervention condition who meet two or more criteria of the set of indicators for proactive palliative care will have additionally regular consultations with a specialized palliative care team. The objectives of the PROLONG study are: 1) to assess the discriminating power of the proposed set of indicators (indicator study) and 2) to assess the effects of proactive palliative care for qualifying patients with COPD on the wellbeing of these patients and their informal caregivers (intervention study). The primary outcome measure of the indicator study is time to death for any cause. The primary outcome measure of the intervention study is the change in quality of life measured by the St George Respiratory Questionnaire (SGRQ) three months after inclusion.

**Discussion:**

The PROLONG study may lead to better understanding of the conditions to start and the effectiveness of proactive palliative care for patients with COPD. Innovative aspects of the PROLONG study are the use of a set of indicators for proactive palliative care, the active involvement of a specialized palliative care team and the use of a patient-tailored proactive palliative care plan.

**Trial registration:**

Netherlands Trial Register (NTR): NTR4037

## Background

In 2002 the World Health Organization (WHO) introduced a new definition of palliative care. They emphasized in this definition the importance of early identification and impeccable assessment and treatment of pain and other problems, physical, psychosocial and spiritual in order to prevent and relief suffering [1]. This means that palliative care is not limited to the terminal phase and can be delivered beside curative care to patients with a life-threatening illness. It implicates that palliative care is not only restricted to reactive symptom relief. By anticipating on expected disease scenario’s and the specific needs and wishes of a patient, problems can be prevented and hence quality of life improves. The clinical use of this proactive palliative care is growing in care for patients with cancer. Still a proactive approach is not very common for patients with Chronic Obstructive Pulmonary Disease (COPD), even though the symptoms that occur in the end stage of COPD are as severe or even worse than in the final stage of lung cancer [2,3]. Both groups of patients prefer a treatment with the emphasis on comfort instead of life prolongation, but to patients with COPD this is offered less frequently [4]. For instance patients with COPD receive less opioids and benzodiazepines than patients with lung cancer for their dyspnea complaints [5], and they die more often at an Intensive Care Unit (ICU) [6].

Several barriers are described with respect to offering proactive palliative care to patients with COPD [7]. A first important barrier is the identification of patients with COPD who can benefit from proactive palliative care, as it is difficult to predict the remaining length of survival of these patients [8]. For that reason, recognizing the appropriate time to start proactive palliative care may not be obvious for clinicians. A second important barrier is the organization of proactive palliative care for COPD patients. The majority of hospitals in Europe have no formalized approach regarding palliative care issues for patients with COPD: these patients have less universal access to specialist palliative care services than those with malignant lung diseases [9-11].

In general, an important problem in the transition or referral to palliative care services is that the term “palliative care” is often associated with terminal or end stage care only. This can be an impediment to early implementation of proactive palliative care as proposed by the WHO. Especially for patients with COPD who do not perceive COPD as an illness that disrupts life [12]. Hence the term ‘supportive care’ may be a term more conducive to referral and may facilitate integration between curative care and palliative care for patients with COPD [13-15]. Therefore, in this study we will speak of supportive care in contact with participating patients with COPD.

### Identification of patients with COPD for proactive palliative care

**Figure 1:**
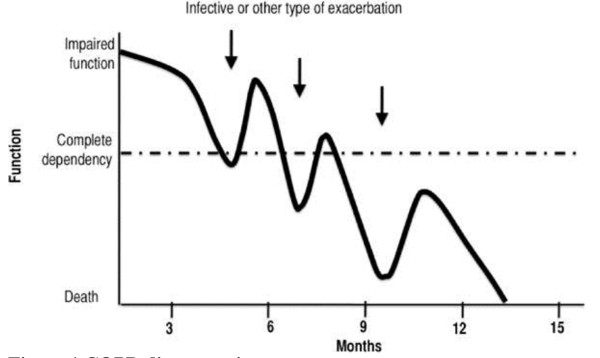
COPD disease trajectory.

COPD illustrates the ‘organ failure’ end-of-life trajectory in which a gradual decline is punctuated by acute severe exacerbations, any one of which may be fatal [16,17]. The patient may survive the majority of these exacerbations as long as he shows resilience and rebounds to (at least part of) his former condition. As it is unclear which exacerbation will be fatal, death may seem to occur suddenly [18] (Figure 1).

This uncertain prognosis makes it difficult for clinicians to initiate discussions about palliative care and end–of–life care planning [19,20]. In stable COPD, population models of 6 month survival do exist but they are of limited value to predict death for individual patients [21]. It is therefore proposed to address proactive palliative care at certain milestones in the course of the disease [22], such as the occurrence of an acute exacerbation COPD (AECOPD) [20]. An AECOPD is defined as ‘an acute worsening of the patient’s condition from the stable state, which is sustained and may warrant the patient to seek additional treatment’ [23]. Exacerbations cluster in time with a high risk of recurrence within 8 weeks of recovery [24], and show an increasing frequency as the disease progresses [25]. Recovery after an AECOPD is often to a suboptimal condition as before the exacerbation and after each exacerbation more care may be required to support the patient and the family. Therefore each admission to the hospital for an AECOPD creates an opportunity to identify patients at high risk of subsequent readmission or post-discharge mortality and hence who can benefit from a proactive palliative care approach. Several studies focused on the identification of predictive factors associated with hospital readmission or mortality for patients with an AECOPD. The findings of these studies are summarized in a recent review [26]. One of the conclusions is that in-hospital mortality is related to the patient’s acute physiological state and to the development of acute comorbidity while post-discharge mortality particularly reflects the severity of the underlying COPD, as well as concomitant specific comorbidities. Important factors influencing the frequency of readmission include functional limitation and poor health-related quality of life. A profile emerges of the types of patients hospitalized for an AECOPD at high risk of subsequent readmission or post-discharge mortality [19].

In accordance with these findings and based on existing literature [19,26-29] we developed a set of indicators for lung specialists to improve the identification of patients hospitalized for an AECOPD for whom proactive palliative care might be beneficial. We hypothesize that the presence of two or more of the following indicators (or prognostic conditions) should be a reason to start proactive palliative care: 1) hypoxaemia or hypercapnia at discharge; 2) treatment of the exacerbation with Non Invasive Ventilation (NIV); 3) patient needs professional home care service for personal care after discharge; 4) a negative answer to the surprise question: ‘Would I (as lung specialist) be surprised if this patient would have a subsequent readmission for AECOPD within 8 weeks and/or would die in the next year?; 5) the diagnosis of a severe comorbidity such as: a) non-curable malignity or b) cor pulmonale (proven or non proven) or c) proven Chronic Heart Failure (CHF) or d) diabetes mellitus with neuropathy or e) renal failure, clearance < 40 Glomerular Filtration Rate (GFR); 6) Clinical COPD Questionnaire (CCQ) total, day version ≥ 3; 7) Medical Research Council dyspnea questionnaire (MRC dyspnea) = 5; 8) Forced Expiratory Volume in 1 second (FEV1), measured before AECOPD < 30% of predicted; 9) Body Mass Index (BMI) < 21 or unplanned weight loss (> 10% weight loss in last 6 months or > 5% in last month); 10) previous hospital admissions for AECOPD (last 2 years ≥ 2 and/or last year ≥ 1); 11) Age > 70 years.

In this study the discriminating power of this set of indicators will be examined. We hypothesize that the set of indicators can predict readmission within 8 weeks and/or death within 1 year for patients hospitalized for an AECOPD.

### Organization of proactive palliative care for patients with COPD

Provision of palliative care for patients with COPD in Europe is variable, and overall very small [11]. The majority of hospitals in Europe do not have a formalized approach to palliative care issues for patients with chronic lung disease. Besides patients with advanced non-malignant respiratory disease have less universal access to specialist palliative care services than those with malignant lung disease [9-11]. In a survey performed in the UK, the minority of hospital units had a formal referral pathway for palliative care and only about 13% had a policy of initiating end-of-life discussions with appropriate patients [30]. Although variation in care may be influenced by many factors including availability, access and reimbursement issues, such geographic variations suggest a lack of consensus concerning the best approach to palliative care for patients with COPD [19].

Therefore, recent studies have focused on the best approach and content of palliative care for patients with COPD. It is suggested to start palliative care early beside curative care [31]. Furthermore, good proactive palliative care should at least consist of: 1) a standardized inventory of current and future care needs and a structured organization of proactive palliative care; 2) advance care planning (ACP), which involves the patient (−family)-clinician communication about end-of-life care and the completion of advanced directives [20]. Important components of ACP are discussions about the expected course of the disease and prognosis and counseling concerning preferences for care at the end of life, including spiritual care [19]; 3) development and implementation of a patient-tailored proactive palliative care plan. There are several problems from the patient as well as from the clinician perspective when it comes to satisfactory implementation of ACP and a patient-tailored proactive palliative care plan [7]. First, there are clinician and patient related barriers to discuss ACP [32]. Patients for instance avoid ACP discussions out of fear of suboptimal treatment in case of emergencies while clinicians are concerned that early ACP will take away patients’ hope [20]. Discussions about ACP are therefore unlikely to occur and when they do occur they are likely to be of poor quality [19]. Second, not all clinicians have a special interest in or are qualified to perform proactive palliative care. Finally, delivering proactive palliative care beside curative care for patients with COPD may increase the workload and clinicians may be faced with shortage of time [7]. In order to overcome these problems it is suggested that a multidisciplinary approach to proactive palliative care with better access to specialist palliative care services will help patients with COPD navigate through the continuum of chronic disease management and will improve quality of end-of-life care [7,10].

Unfortunately, no research data is available on the beneficial effects of a multidisciplinary approach to proactive palliative care for patients with COPD in terms of reducing the healthcare utilization (for example, hospital readmission) or improving quality of life. However, in a study in male patients predominantly diagnosed with cancer but also with cardiovascular and pulmonary diseases, the benefits of palliative care provision appeared effective compared with usual care [33]. Patients receiving palliative care were less likely to be admitted to the ICU during hospitalization, had lower inpatient cost per day and received better medical care provision compared to usual care patients. Hence proactive palliative care may avoid admission to the ICU for patients with COPD and may help to reduce health care costs. More research data is available concerning the beneficial effects of an early introduction of palliative care for patients with cancer. In a recent study [34] the effect of introducing early palliative care among patients diagnosed with metastatic non-small-cell lung cancer was examined in a randomized controlled trial (RCT). As compared to patients receiving standard care, patients receiving early palliative care had a better quality of life, less depressive symptoms, less aggressive care at the end of life and longer survival.

In the present study the effects of proactive palliative care performed by a specialized palliative care team for patients with COPD on the wellbeing of these patients and their informal caregivers will be examined. We hypothesize that proactive palliative care for patients with COPD will: increase the quality of life of these patients, decrease the number and length of acute hospital admissions and ICU admissions, prolong survival of these patients, decrease the number of patients that die in the ICU, and decrease the level of overburdening of their informal caregivers.

## Methods/Design

### Objectives

The PROLONG study exists of two parts, an indicator study and an intervention study, each with its own primary and secondary objectives:

#### Objectives indicator study

1. The primary objective is to assess the discriminating power of a set of indicators that indicates the start of proactive palliative care for patients with COPD.

2. The secondary objective is to examine to what extend individual indicators (or clusters) indicative are for the need of proactive palliative care.

#### Objectives intervention study

1. The primary objective is to assess the effects of proactive palliative care delivered by a specialized palliative care team on the wellbeing of patients with COPD with poor prognosis and their informal caregivers.

2. The secondary objective: is to assess survival rate in COPD patients with proactive palliative care integrated with standard care versus standard care only.

### Study design

The study consists of a controlled trial (assessment) with hospital as cluster, with a pre- and a post-test assessment. In total 6 hospitals will participate, 3 hospitals in the intervention condition and 3 hospitals in the control condition. Hospitals are selected for the intervention condition based on the presence of a specialized palliative care team. In the hospitals in the control condition standard care (usual care) will be delivered to patients with COPD by their treating lung specialists. In the hospitals in the intervention condition all patients with COPD will receive standard care by their treating lung specialist and those patients that are indicated for proactive palliative care by our set of indicators will also be supported by a specialized palliative care team on a regular base. Baseline measurements of the intervention study will be assessed from all participating patients during hospitalization for AECOPD before start of the intervention. Follow-up measurements will take place every three months, starting from the moment of discharge for a period of one year or until death. The primary outcome measure of the indicator study is time to death for any cause. The primary outcome measure of the intervention study is the change in quality of life measured by the St George Respiratory Questionnaire (SGRQ) at three month after baseline. As pre-test assessment, data will be obtained from the databases of the participating hospitals over a one year period preceding the assessment. These data on hospital-level are necessary to be able to compare hospitals at baseline. As post-test assessment, retrospectively the medical files of all participating patients will be examined over the assessment period. The assessment will take 18 month; 6 month for inclusion and 12 months for follow-up. The post-test assessment will be performed in the 3 months after the assessment.

### Study population

Patients with a hospital admission for AECOPD will be invited to participate. If they agree to participate, their main informal caregiver will also be asked to participate.

#### Inclusion criteria

In order to be eligible to participate, a patient must meet the following criteria:

● Being admitted to the hospital for AECOPD, and

● Aged 18 years or older.

#### Exclusion criteria

A patient that meets any of the following criteria will be excluded from participation:

● Not speaking the Dutch language, or

● Having severe cognitive disorders, or

● At moment of inclusion being treated by a specialized palliative care team.

### Intervention

In the hospitals in the intervention condition, members of the specialized palliative care teams will receive a special training in the provision of proactive palliative care for patients with COPD. The training will be provided by academic palliative care professionals of the Radboudumc in Nijmegen. These trainings consist of two consecutive meetings of three hours each. The first meeting will take place in the month before start of the assessment. The second meeting will take place in the first month of the assessment. The following topics will be discussed:

● How to communicate end of life aspects with patient and family;

● How to create a patient-tailored proactive palliative care plan;

● How to anticipate on illness- and dying scenarios proactively;

● How to organize transfer of care to lung specialist and general practitioner (GP);

● How to perform a proactive palliative care plan in cooperation with the lung specialist.

During the controlled trial patients in the intervention condition who are assigned for proactive palliative care will meet with a member of the specialized palliative care team within one week after enrollment and at least monthly thereafter in the outpatients setting for at least one year or until death. The main informal caregiver of the patient will be asked to be present at those meetings. Guidelines for the proactive palliative care meetings in the ambulatory setting are adapted from the general guidelines palliative care in the Netherlands [35].

### Study parameters

#### Outcome measures indicator study

1. Primary outcome measures:

a. Length of time from the moment that a patient hospitalized for AECOPD meets two or more criteria of the set of indicators to death for any cause.

2. Secondary outcome measures:

a. Length of time from the moment that a patient hospitalized for AECOPD meets two or more criteria of the set of indicators to the first unexpected readmission to the hospital for AECOPD.

b. The sensitivity and specificity of the set of indicators, that indicate the start of proactive palliative care for patients hospitalized for COPD, in predicting death for any cause within 1 year.

c. The sensitivity and specificity of the set of indicators, that indicate the start of proactive palliative care for patients with COPD, in predicting the first unexpected readmission to hospital for AECOPD within 8 weeks.

d. Length of time from the moment that a patient hospitalized for AECOPD meets two or more criteria of the set of indicators to death as a result of pulmonary insufficiency.

e. The sensitivity and specificity of the set of indicators, that indicate the start of proactive palliative care for patients hospitalized for COPD, in predicting death as a result of pulmonary insufficiency within 1 year.

f. The contribution of individual indicators (or clusters), in predicting death for any cause within 1 year.

g. The contribution of individual indicators (or clusters), in predicting the first unexpected readmission to hospital for AECOPD within 8 weeks.

h. The contribution of individual indicators (or clusters), in predicting death as a result of pulmonary insufficiency within 1 year.

#### Outcome measures intervention study

1. The primary outcome measure is:

a. Change in quality of life (St George Respiratory Questionnaire (SGRQ) [36]) of the patient 3 months after inclusion

2. The secondary outcome measures are:

Patient-related

a. Change in quality of life (SGRQ) of the patient 6, 9 and 12 months after inclusion

b. Change in quality of life at the end of life (McGill Quality of Life questionnaire (McGill QOL) [37]) 3, 6, 9 and 12 months after inclusion

c. Change in psychological wellbeing (Hospital Anxiety and Depression Scale (HADS) [38]) 3, 6, 9 and 12 months after inclusion

d. Change in illness understanding 3, 6, 9 and 12 months after inclusion

e. Number and length of unexpected hospital admissions

f. Number and length of unexpected ICU admissions

g. Are the choices of Advance Care Planning (ACP) documented in the medical file? (when yes/when no)

h. Place of death (ICU/hospital/hospice/nursing home/at home)

i. Is preferred place of death known? (when yes/when no)

j. Has this wish come true? (when yes/when no)

k. Length of survival of COPD patients with proactive palliative care integrated with standard care versus standard care only

Informal caregiver-related

a. Change in informal caregiver burden (Self-Perceived Pressure from Informal Care questionnaire (SPPIC) [39]) 3, 6, 9 and 12 months after inclusion

b. Change in psychological wellbeing (HADS) at 3, 6, 9 and 12 months after inclusion

c. Change in illness understanding at 3, 6, 9 and 12 months after inclusion

#### Other study parameters

In order to take account of possible confounding variables, other parameters are: age, gender, marital status, socio-economic status, smoking history, condition of living (single, or living together), and place of living (home, residential home, or nursing home).

### Randomization

Randomization will not take place. Hospitals will be selected for the intervention condition based on the presence of a specialized palliative care team in the hospital. In order to be able to compare the hospitals in the control- and the intervention condition a pre-test assessment will be performed.

### Study procedure

A description of the procedure is given to assess the defined study parameters.

#### Pre-test assessment

The following data on hospital level will be obtained from the databases of the participating hospitals retrospectively over a period of one year (1-1-2013 till 1-1-2014):

● Number of hospitalizations for AECOPD (including ICU admissions)

● Number of unique patients hospitalized for AECOPD (including ICU admissions)

● Total number of days of hospitalization of patients with an AECOPD (including ICU admissions)

● Number of hospitalizations in the ICU (exclusively) for AECOPD

● Number of unique patients hospitalized for AECOPD in the ICU (exclusively)

● Total number of days of hospitalization of patients with an AECOPD in the ICU (exclusively)

● Total number of patients hospitalized for AECOPD that have died in the hospital (in the ICU or on the nursing unit)

#### Training

**Figure 2:**
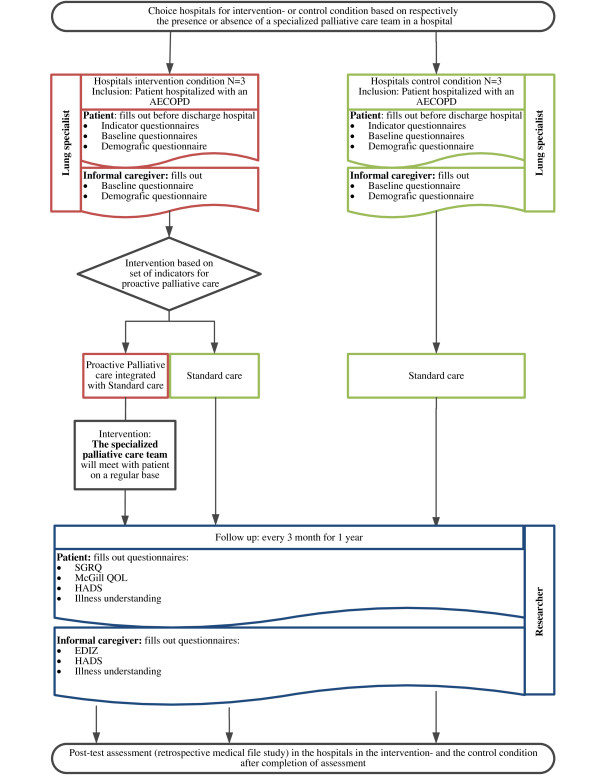
Study schema of the controlled trial (assessment) and the post-test assessment for PROLONG.

Before start of the controlled trial (see Figure 2), members of the specialized palliative care teams in the hospitals in the intervention condition will receive a special training in provision of proactive palliative care for patients with COPD.

#### Controlled trial (assessment)

##### Patient

Patients in the control condition will receive standard care (usual care) only. Patients in the intervention condition will receive standard care and, only if they meet two or more criteria of the set of indicators for proactive palliative care they will receive proactive palliative care integrated with standard care. The procedure in the control condition will be first described before describing the procedure in the intervention condition.

In the hospitals in the control condition eligible patients will be recruited the second day after hospital admission. The treating lung specialist will give each eligible patient oral and written information about the study. The written information consists of an information leaflet and two informed consent forms: one for the patient and one for the informal caregiver. In the information leaflet a description of the study, including the nature of participation and phone numbers for study contacts, are given for the patient and the informal caregiver. The lung specialist will obtain written informed consent from the patient prior to enrollment. If a patient has consented, a lung nurse will distribute questionnaires for completion on the sixth day of hospital stay or in case the patient is discharged earlier on the day before leaving the clinic. After hospital discharge the patient will be asked by the study coordinator to complete questionnaires every 3 month until the end of the study or until death. The questionnaires will be sent to the patient by mail. A stamped retour envelope will be enclosed.

In the hospitals in the intervention condition the same procedure will be followed as in the hospitals in the control condition. In addition lung specialists will check if a patient meets two or more criteria of the set of indicators for proactive palliative care on the sixth day of hospital admission or in case the patient is discharged earlier on the day before leaving the clinic. If so, the lung specialist will inform the specialized palliative care team. Patients who are assigned for proactive palliative care will preferably meet for the first time with a physician of the specialized palliative care team before being dismissed from the hospital. If this is not possible the first meeting will take place within 1 week after enrolment. Thereafter, the specialized palliative care team will meet with the patient monthly in the outpatients setting for at least 1 year or until death.

##### Informal caregiver

During hospital stay, each eligible patient will identify a main informal caregiver, a relative or friend whom he or she relies upon most for help. By sharing the written information with the informal caregiver, the patient will provide the informal caregiver with information about the study. The informal caregiver can only participate if the patient is also participating. Once the informal caregiver has consented questionnaires will be distributed during hospital stay and every 3 month after hospital discharge of the patient. These questionnaires will be sent in the same envelop as the patient questionnaires.

##### Lung specialist

The treating lung specialist will provide standard care (usual care) to the patients hospitalized for an AECOPD. After the patient has filled out and returned the questionnaires the lung specialist in the control- and the intervention condition will fill out a case report form (CRF). The CRF consists of questions about the measurement results of each indicator of the set of indicators. The set of indicators is represented in Table 1. Only in the intervention condition the lung specialist will check if a patient meets two or more criteria of the set of indicators for proactive palliative care.

**Table 1:** Set of indicators for proactive palliative care

**A patient hospitalized for AECOPD is eligible for proactive palliative care when meeting two or more criteria of the following set of indicators:**	
1.	Hypoxaemia or hypercapnia at discharge
2.	Treatment of the exacerbation with Non Invasive Ventilation (NIV)
3.	Patient needs professional home care service for personal care after discharge
4.	Negative answer to the surprise question: ‘Would I (as lung specialist) be surprised if this patient would have a subsequent readmission for AECOPD within 8 weeks and/or would die in the next year?
5.	The diagnosis of a severe comorbidity such as:
	a. Non-curable malignity or
	b. Cor pulmonale (proven or non proven) or
	c. Proven Chronic Heart Failure (CHF) or
	d. Diabetes mellitus with neuropathy or
	e. Renal failure, clearance < 40 (GFR: in ml/min)
6.	CCQ total, day version ≥ 3
7.	MRC dyspnea = 5
8.	FEV1 (measured before AECOPD) < 30% of predicted
9.	BMI < 21 or unplanned weight loss (> 10% weight loss in last 6 months or > 5% in last month)
10.	Previous hospital admissions for AECOPD (last 2 years ≥ 2 and/or last year ≥ 1)
11.	Age > 70 years

##### Specialized palliative care team

The specialized palliative care teams in the hospitals in the intervention condition consist of specially trained teams of professionals who provide care and support in inpatient and outpatient settings. A team consists of at least a physician who is specialized in palliative care, a nurse who is specialized in palliative care, and preferably a psychologist and a spiritual counselor.

#### Post-test assessment

A post-test assessment will be performed in all participating hospitals. The medical files of all participating patients will be examined retrospectively regarding the assessment period on the following measures:

● Number and length of hospitalization of unexpected hospital admissions for AECOPD

● Number and length of hospitalization of unexpected ICU admissions for AECOPD

● Are the choices of Advance Care Planning (ACP) documented in the medical file? (when yes/when no)

● Place of death (ICU/hospital/hospice/nursing/home/at home)

● Is preferred place of death known? (when yes/when no)

● Has this wish come true? (when yes/when no)

● Length of survival after meeting conditions for proactive palliative care

● The primary and secondary measures of the indicator study

In order to take account of possible confounding variables, other parameters are: age, gender, marital status, type of admission, (acute or planned), condition of living (single, or living together), place of living (home, residential home, or nursing home), Gold stage, comorbidities, and date of diagnosis.

### Tools to help the clinical decision making

In order to help the clinical decision making, the specialized palliative care team will make use of two additional tools: 1) the Problems and Needs in Palliative Care questionnaire short version (PNPC-sv) and 2) the Proactive Palliative Care Planning Card (PPCPC).

The PNPC-sv patient is a concise, patient-centered tool that helps to identify the problems affecting the patient’s quality of life and (unmet) needs for care. This self-report questionnaire is covering all dimensions of palliative care. The questionnaire consists of 36 items and is a reliable and valid tool [40].

The PPCPC is a tool that can be used by members of the specialized palliative care team to structure the discussion with the patient and his/her informal caregiver. This tool is especially useful when exploring the actual en potential problems and needs of the patient [41].

### Data collection

Data collection will take place by administration of questionnaires to the participating patients and their informal caregivers and by retrospectively collecting data from the medical files of the participating patients over the assessment period. The type of questionnaires for patients and informal caregivers, the frequency of their administration and the retrospectively collected data are detailed in Table 2.

**Table 2:** Overview of outcome measures per time point in the PROLONG study

**Outcome measures**	**B**	**3 m**	**6 m**	**9 m**	**12 m**	**R**
**Questionnaires**						
*Patient*						
CCQ	X					
MRC dyspnea	X					
SGRQ	X	X	X	X	X	
McGill QOL	X	X	X	X	X	
HADS	X	X	X	X	X	
Illness understanding	X	X	X	X	X	
Demographic questionnaire	X					
*Informal caregiver*						
SPPIC	X	X	X	X	X	
HADS	X	X	X	X	X	
Illness understanding	X	X	X	X	X	
Demographic questionnaire	X					
*Lung specialist*						
CRF	X					
**Medical files**						
Number of hospitalisations of unexpected hospital admissions for AECOPD						X
Number of days of unexpected hospital admissions for AECOPD						X
Number of hospitalisations of unexpected ICU admissions for AECOPD						X
Number of days of unexpected ICU admissions for AECOPD						X
Are the choices of ACP documented in the medical file at baseline? (when yes/when no)						X
Are the choices of ACP documented in the medical file after one year or at time of death? (when yes/when no)						X
Did the patient die within one year after inclusion? (when yes/when no)						X
Date of death						X
Place of death (ICU/hospital/hospice/nursing home/at home)						X
Is preferred place of death known? (when yes/when no)						X
Has this wish come true? (when yes/when no)						X
Primary cause of death (pulmonary insufficiency/other cause)						X
Secondary cause of death (pulmonary insufficiency/other cause)						X
Did the patient have an unexpected hospital readmission for AECOPD within 8 weeks? (when yes/when no)						X
Date of first unexpected hospital readmission for AECOPD.						X

B = baseline; m = month; R = retrospectively.

### Instrument selection

#### Questionnaires used as indicator

In order to be able to decide whether or not to start proactive palliative care, questionnaires used as indicator will be filled out by the patient.

We will use the CCQ day version to measure health status of patients. The CCQ is a questionnaire for self-administration specially developed to measure health status in patients with COPD and is valid, responsive, and reliable [42,43]. The CCQ consists of 10 questions rated on a seven point Likert scale. Higher scores represent a worse health status. Questions are divided into three domains: symptoms (4 questions), functional status (4 questions), and mental state (2 questions).

The MRC dyspnea scale has been in use for many years for grading the effect of breathlessness on daily activities [44]. The MRC dyspnea scale consists of 5 questions and provides a simple and valid method of categorizing patients in terms of their disability due to COPD [44]. The patient’s dyspnea is rated from 1–5 in terms of severity, with the higher the grade, the more severe the dyspnea. During the study MRC dyspnea will be measured by asking about the circumstances two weeks before hospital admission.

#### Questionnaires used as outcome measures

##### Patient questionnaires

The SGRQ is a specific quality of life questionnaire for obstructive respiratory diseases [36]. It consists of 50 questions from which a total score is calculated. It is divided into three subscales: symptoms (8 items related to patients’ recollection of their symptoms), activities (16 items on physical activities which are caused or limited by dyspnea), and impacts (26 items on the social and physiologic effects of the disease). The final score obtained ranges from zero to 100. A higher score indicates a lower quality of life. A score change of 4 points or more is considered significant in the quality of life of the patient [45]. The SGRQ is a reliable and valid measure of the quality of life in patients with COPD [46].

The McGill QOL is designed to assess quality of life in patients with a life-threatening illness [37]. The questionnaire consists of 16 items with an 11-point scale (0–10) with appropriate anchors. It includes 5 domains: physical symptoms, physical well-being, psychological well-being, existential issues and support. The mean of all 5 domains is presented as McGill QOL total score. The acceptability, internal consistency, reliability and validity of the McGill QOL have been assessed in patients receiving palliative care [47].

The HADS will be used to assess psychological wellbeing in patients. The HADS is a self-assessment 14-item questionnaire. It has two 7-item subscales assessing depression and anxiety in the preceding week. The format consists of four answering categories (0–3) that quantify the degree to which a particular emotion is experienced by the patient. The score on each subscale ranges from 0 to 21 and a score larger than 11 is considered to be consistent with definitive depression and anxiety. A score less than 7 is normal and a score of 8–10 is considered borderline for depression and anxiety [38].

Patients with COPD tend to be poorly informed about the long-term prognosis of COPD and what to expect toward the end of life [48]. They may not realize that COPD is incurable and fatal. They also may not always attribute repeated exacerbations to advancing disease but instead seeing them as temporary setbacks caused by activities, environmental factors, faltering self-management, or infection. Toward the end of life this lack of understanding may impair quality of life [2,49]. There are no validated tools to assess illness understanding in patients with COPD. Therefore, we adapted an illness understanding questionnaire used in studies of patients with advanced cancer [50]. The questionnaire consists of 4 self-report items which can be answered by yes or no.

Patients will be asked in a demographic questionnaire to indicate their sex, age, marital status, education, smoking history, living situation, name of main informal caregiver and kind of relationship with their main informal caregiver.

##### Informal caregiver questionnaires

The Self-Perceived Pressure from Informal Care questionnaire (SPPIC) is a non-disease specific instrument assessing the demands of the informal caregiver situation [39]. This instrument consists of nine statements that form a hierarchical scale that varies from less to more pressure. The statements are all related to the subjective perception of the informal caregiver. It is a validated and easy to use instrument since completing it takes less than 5 minutes.

The HADS (see patient questionnaires) will also be used in informal caregivers to assess psychological wellbeing. An adapted version of the illness understanding questionnaire for patients will be used for the informal caregivers as well. Finally, informal caregivers will be asked to indicate their sex, age and education in a demographic questionnaire.

### Sample size calculation

The primary outcome in the intervention study is the quality of life of the patients measured with the SGRQ. More specifically the change in SGRQ three month after inclusion is the variable of interest. Koff et al. [51] published a difference in change of 9 between the two groups (standard care, standard care with proactive palliative care) with a common standard deviation of the change of 16. Then 64 patients would be needed in each group to obtain a power of 80% (two-sided *t*-test, alpha = 0.05). To adjust for the clustering at hospital level (ICC = 0.01, three hospitals per arm) and to allow for an additional loss to follow up of 10% a total of 86 patients are needed in each arm. This means that in each hospital 29 patients with an AECOPD are needed who have a poor prognosis according to our criteria. We expect to have to include between 60 and 90 patients with an AECOPD in each hospital to get sufficient patients that meet at least 2 criteria of the set of indicators.

### Statistical analysis

#### Study parameter(s) indicator study

Non-continuous data will be reported as frequencies. Continuous variables normally distributed will be reported as mean ± standard deviation (SD). Not normally distributed data will be reported as median (interquartile range, IQR). The analytical plan consists of two steps. The first step will be to explore the discriminating power of meeting two or more criteria of the set of indicators for predicting death within one year and predicting unexpected hospital admission, respectively. The sensitivity and specificity for both death within one year and for unexpected admission to hospital will be presented. The second step will be to explore the discriminating power of individual indicators (or clusters) in predicting death within one year or unexpected admission to hospital. Univariable and multivariable logistic regression will be performed to examine which variables or cluster of variables are associated with death within one year (unexpected admission to hospital respectively). Variables will be eliminated one by one from the model based on likelihood ratio tests. Variables are eligible for inclusion into the final model if they are significantly associated with death within one year (unexpected admission to hospital respectively), with a p-value of < 0.10.

#### Study parameter(s) intervention study

Frequencies, means and standard deviations will be used to describe the study variables. Differences between the study groups in baseline characteristics and clinical outcomes will be assessed and tested for statistical significance with the use of two-sided Fisher’s exact tests and chi-square tests for categorical variables and independent-samples t-tests for continuous variables. The primary study outcome measure of the intervention study is the change in the score on the SGRQ from baseline to 3 months: a paired *t*-test will be used to test the difference between the groups for statistical significance. Linear mixed models, with adjustment for baseline scores, will be used to study the effect of proactive palliative care on SGRQ outcomes during the follow-up period. The analysis follows the principle of intention to treat. Survival time will be calculated from the date of enrollment to the date of death with the use of the Kaplan-Meier method. A Cox proportional-hazard model will be used to assess the effect of proactive palliative care on survival, with adjustment for demographic characteristics.

### Ethical considerations

Patients with COPD who participate in the study may be vulnerable but are certainly capacitated adults. Since this is a therapeutic research the participants, patients and informal caregivers, may even benefit from participation. The potential risks of this study are quite small. They relate to the burden of filling in questionnaires. This will take the patient about 20–30 minutes every three months. The potential benefits on the other hand are comparatively large. First, participating patients with severe COPD and their informal caregivers in the intervention condition will get, if indicated for it, extra proactive palliative care. Second, this study may lead to better understanding of the conditions to start and the effectiveness of proactive palliative care for patients with COPD. Third, there is a potentially benefit for society since results of this study may ultimately lead to different and improved clinical approaches to care of patients with severe COPD.

This Study has been approved by the Medical Ethics Committee (CMO) of the Radboud University Nijmegen Medical Centre (METC protocol number 2012/260).

## Discussion

Research on the effectiveness of palliative care for the COPD patient is scarce and inconclusive [52]. The PROLONG study is the first prospective controlled trial evaluating the effectiveness of a multidisciplinary approach to palliative care in COPD disease. The outcomes of this study will give insight in the discriminating power of a set of indicators for proactive palliative care and the effectiveness of proactive palliative care for patients with COPD.

In the Netherlands, COPD is on the sixth place of causes of death for people older than 45 years. In 2011 6,535 patients died with COPD as primary cause of death while in 2010 the number of hospitalizations of patients with COPD as diagnosis was 22,5440. The prevalence of COPD in the Netherlands is high (361,800 in 2011) and will, with the aging of the population, further increase the coming years [53]. Up to now patients with COPD hardly receive palliative care. The above mentioned numbers reveal the social relevance of introducing palliative care for patients with COPD. Furthermore, introducing palliative care for patients with COPD can possibly be cost-saving since palliative care may lead to reduction of acute care [33,54,55].

We have chosen a cluster controlled design for the PROLONG study to prevent cross-contamination of the intervention within a hospital. At the moment of recruitment of hospitals the minority of hospitals in the Netherlands had the availability of a specialized palliative care team. Therefore, it was not an option to perform a randomized cluster controlled trial. Hospitals are selected for the intervention condition based on the presence of a specialized palliative care team in the hospital. In order to be able to compare the hospitals in the control- and the intervention condition at baseline a pre-test assessment will be performed.

Recruitment for trials of patients with poor prognosis is often difficult. In order to ensure a sufficiently large sample for the PROLONG study broad inclusion criteria will be used. All patients hospitalized with an AECOPD over 17 years old can be screened for the study. This will facilitate lung specialists to include patients. Only after inclusion the distinction between patients with poor or better prognosis will be made in order to decide who is eligible for the intervention condition.

The PROLONG study may lead to better understanding of the conditions to start and the effectiveness of proactive palliative care for patients with COPD. The innovative aspects of the PROLONG study are: 1) the use of a set of indicators to identify patients hospitalized with an AECOPD who are in need of proactive palliative care, 2) the active involvement of a specialized palliative care team in the development and the implementation of proactive palliative care for patients with COPD and, 3) the use of a patient-tailored proactive palliative care plan in which lung specialist and a specialized palliative care team work together to optimize proactive palliative care for the COPD patient. This patient-tailored proactive palliative care plan is intended to meet and to adjust to the individual needs, wishes, possibilities and limitations of the patient and the informal caregiver.

## Abbreviations

ACP: Advance care planning; AECOPD: Acute exacerbation chronic obstructive pulmonary disease; BMI: Body mass index; CCQ: Clinical COPD questionnaire; CHF: Chronic heart failure; COPD: Chronic obstructive pulmonary disease; CRF: Case report form; FEV1: Forced expiratory volume in 1 second; GP: General practitioner; GRF: Glomerular filtration rate; HADS: Hospital Anxiety and Depression Scale; ICU: Intensive care unit; IQR: Inter quartile range; MRC dyspnea: Medical Research Council dyspnea questionnaire; MUST: Malnutrition Universal Screening Tool; NIV: Non-invasive ventilation; PNPC-sv: Problems and needs in palliative care questionnaire short version; PPCPC: Proactive palliative care planning card; QOL: Quality of life; RCT: Randomized controlled trial; SD: Standard deviation; SGRQ: St George Respiratory Questionnaire; SPPIC: Self-perceived pressure from informal care questionnaire; WHO: World Health Organization.

## Competing interests

The authors declare that they have no competing interests that are directly relevant to the content of this article.

## Authors’ contributions

RGD led the drafting of this paper and was together with YE, YH and SV responsible for the development of the protocol. All authors contributed to the design of the study. YE, YH and KV were the initiators of the study and obtained funding. YE, YH and SV did study supervision. YE, YH, SV, RD and KV were responsible for critical revision of the manuscript. All authors read, revised and approved the final manuscript.

## Authors’ information

RGD is the principal investigator in this study. YH is lung specialist, associate professor of Pulmonology and leads the research performed in the field of COPD at the Department of Lung Diseases of the Radboud University Nijmegen Medical Centre. She is President of the board of the Dutch Thoracic Society (NVALT). SV is oncologist, medical consulent palliative care and staff member of the Department of Medical Oncology and the Department of Pain and Palliative Medicine of the Radboud University Nijmegen Medical Centre. RD is lung specialist, professor of Pulmonology and head of the Academic Center of Lung Diseases of the Radboud University Nijmegen Medical Centre. He chairs the Dutch guidelines on the treatment of COPD and is chairman of the Heart-Lung Centre of the Radboud University Nijmegen Medical Centre. KV is anesthesiologist, professor in Pain and Palliative Medicine and chairman of the Academic Center of Pain and Palliative Medicine of the Radboud University Nijmegen Medical Centre. He is President Elect and member of the Executive Board of the World Institute of Pain and Honorary Secretary of the Benelux Chapter of the World Institute of Pain. YE is assistant professor at the Department of Pain and Palliative Medicine of the Radboud University Nijmegen Medical Centre. She combines experience in quality of care research (indicator development and implementation, improving quality of care, changing behavior of professionals) with experience in research in pain and palliative care.

## Pre-publication history

The pre-publication history for this paper can be accessed here:

http://www.biomedcentral.com/1471-2466/14/54/prepub
